# RCRFNet: Enhancing Object Detection with Self-Supervised Radar–Camera Fusion and Open-Set Recognition

**DOI:** 10.3390/s24154803

**Published:** 2024-07-24

**Authors:** Minwei Chen, Yajun Liu, Zenghui Zhang, Weiwei Guo

**Affiliations:** 1 Shanghai Key Laboratory of Intelligent Sensing and Recognition, Shanghai Jiao Tong University, Shanghai 200240, China; chenminwei2022@sjtu.edu.cn (M.C.); liuyajun@sjtu.edu.cn (Y.L.); 2 Center of Digital Innovation, Tongji University, Shanghai 200092, China; weiweiguo@tongji.edu.cn

**Keywords:** radar–camera fusion, target detection, self-supervised learning, open-set recognition, autonomous driving

## Abstract

Robust object detection in complex environments, poor visual conditions, and open scenarios presents significant technical challenges in autonomous driving. These challenges necessitate the development of advanced fusion methods for millimeter-wave (mmWave) radar point cloud data and visual images. To address these issues, this paper proposes a radar–camera robust fusion network (RCRFNet), which leverages self-supervised learning and open-set recognition to effectively utilise the complementary information from both sensors. Specifically, the network uses matched radar–camera data through a frustum association approach to generate self-supervised signals, enhancing network training. The integration of global and local depth consistencies between radar point clouds and visual images, along with image features, helps construct object class confidence levels for detecting unknown targets. Additionally, these techniques are combined with a multi-layer feature extraction backbone and a multimodal feature detection head to achieve robust object detection. Experiments on the nuScenes public dataset demonstrate that RCRFNet outperforms state-of-the-art (SOTA) methods, particularly in conditions of low visual visibility and when detecting unknown class objects.

## 1. Introduction

Autonomous driving technology has seen rapid advancements in recent years, focusing mainly on three critical components: environment perception, decision-making, and control [1]. Environment perception is the foundation of autonomous driving, performing functions such as 2D and 3D object detection, depth estimation, and prediction, similar to how human eyes work. This perception relies on real-time data gathered by onboard sensors. However, the data obtained from a single sensor are often insufficient for the complex tasks required for environment perception.

Common onboard sensors include vision cameras, LiDAR, and millimeter-wave (mmWave) radar. Vision cameras, while providing rich geometric information, are significantly impacted by lighting conditions and have a reliable detection distance limited to 50 m. LiDAR sensors provide high-resolution 3D mapping and precise distance measurements, making them excellent for detailed environmental modelling, but they are costly and less effective in bad weather [2]. In contrast, mmWave radar emits active signals to measure the reflective information of objects. Although it offers less geometric detail, it provides a reliable detection distance of up to 250 m and has strong penetrability, making it ideal for harsh environments. Consequently, mmWave radar has become an indispensable choice for current automated driving applications.

Adverse weather conditions, including heavy rain and fog, present significant challenges to visual detection technology, leading to reduced object detection performance in models such as SSD [3], DETR [4], and Faster R-CNN [5]. Additionally, the diversity of objects in real-world scenes often surpasses the categories covered by detection model training data, resulting in a model mismatch problem [6]. Recently, there has been increasing interest in open-set detection and recognition methodologies [7,8]. In [9], a deep learning approach is proposed for open-set recognition, which involves fitting extreme value distributions specific to each category. This method utilizes the class probabilities derived from these distributions to identify open-set images. In the realm of video action recognition, ref. [10] introduces deep evidence learning grounded in evidence theory, effectively addressing classification uncertainty in video actions. Moreover, ref. [11] introduces a reconstruction learning algorithm aimed at enhancing the robustness of detecting unknown classes.

Radar can reliably detect a wide range of object categories, including those not present in the training set, making it useful for detecting obstacles outside the training categories. As a result, the fusion of radar and camera technologies has gained considerable attention in recent years, aiming to leverage the strengths of both sensors. Millimeter-wave radar point clouds offer precise measurements of object distance and velocity [2]. However, due to their low angular resolution and sparsity, they cannot independently perform complex object detection tasks or accurately identify an object’s category and geometric information. Traditional mmWave radar point cloud processing methods, such as distance-FFT, Doppler-FFT, incoherent accumulation, and angle estimation, have limitations in achieving comprehensive object detection. Recent advancements include Radar-pointGNN [12], which uses graph neural networks for object detection, and methods that utilise raw radar tensor data for 3D object detection through graph neural networks [13]. However, in order to extract features, these algorithms largely rely on large amounts of training data and prior knowledge. This poses difficulties when fusing with image data, which results in reduced robustness and increased complexity. Consequently, radar and vision machine learning fusion detection methods have gained increasing attention from researchers. These approaches aim to integrate the strengths of both radar and vision systems to enhance detection capabilities and overcome the limitations of individual sensor modalities.

Currently, there are three primary approaches for multimodal fusion [14]: early fusion (data-level) methods [15], intermediate fusion (feature-level) methods [16,17,18,19], and late fusion (decision-level) methods [20,21]. In data-level fusion, the region of interest (ROI) is first generated based on radar points [22]. Subsequently, the corresponding region in the visual image is extracted using this ROI. Finally, object detection is conducted on these images using a feature extractor and classifier. For instance, in [23], a fusion of the mmWave radar and camera vision is proposed for pedestrian tracking, whereby the size of the initial ROI is determined by the distance between the obstacle and mmWave radar. In [24], Kadow et al. applied the Haar-like model for feature extraction, which is a classic feature extraction algorithm for face detection. Data-level fusion operates at the most basic level of information, making it vulnerable to uncertainties, incompleteness, and instability in sensor data [25]. Feature-level fusion methods, offering greater flexibility, typically involve layer-by-layer feature extraction and fusion. For instance, in [26], mmWave radar is integrated into visual detection models using frustum correlation to associate heterogeneous data sources. In [27], the authors proposed a transformer-based approach for LiDAR–camera fusion, enhancing robustness against degraded images and sensor misalignment through soft correlation strategies. In [18], to tackle issues such as low angular resolution in mmWave radar, which complicates distinguishing radial objects and leads to false-positive ghost points due to multi-path interference, radar data are augmented with image data containing semantic features. These augmented radar data are then fused by transforming the prediction frame of image modalities into polar coordinate systems. The paper [28] proposes a Spatial Attention Fusion (SAF) module for sensor feature fusion, demonstrating that fusing mmWave radar point clouds significantly enhances detection robustness and improves performance in various weather conditions. Decision-level fusion involves each sensor processing its data independently and making decisions, followed by transmitting the results to a fusion centre for final decision-making; this includes two steps: sensing information processing [29] and decision fusion [30]. For instance, some researches have utilised lists of radar detection targets to validate the results of vision-based detection [31]. Additionally, reference [30] introduced the motion stereo algorithm to further adjust and refine the final detection outcomes. Mainstream traditional methods for decision-level fusion include D-S evidence theory, Bayesian reasoning, and fuzzy inference theory, among others. Although most fusion detection models demonstrate improved accuracy compared to single-sensor models, they still encounter difficulties in complex environments, poor visual conditions, and open scenarios.

To address the aforementioned challenges, this paper presents a novel radar–camera fusion detection method. The key contributions are as follows:

(1) Development of a new fusion detection framework with parallel inputs: This framework enhances feature extraction from visual images and radar point clouds through multiple modules. It aims to generate effective data features for robust detection, even in adverse weather conditions like heavy rain and fog.

(2) Introduction of a novel data correlation and self-supervised learning technique: This method effectively integrates radar point clouds and visual images, leveraging their complementary strengths to enhance the model’s robustness. It incorporates an attention mechanism and self-supervised learning for efficient fusion.

(3) Implementation of an unknown category recognition method with confidence assessment: This approach utilizes depth estimation from radar point clouds and visual images to improve the detection and recognition of open-set categories. By leveraging depth information, the model enhances its capability to identify unknown categories while also improving the classification of known categories.

The rest of this paper is organised as follows. Section 2 presents the fusion detection framework, emphasizing robust feature extraction, radar–camera correlation, self-supervised learning, and open-set recognition. Section 3 conducts experimental validation and comparative analyses. Section 4 provides the ablation study of each proposed module. Finally, Section 5 concludes the entire paper.

## 2. Materials and Methods

### 2.1. Overall Network Structure

To enhance object detection performance in low-visibility and open conditions, we propose a novel deep learning-based radar camera robust feature fusion network (RCRFNet). This network effectively leverages the complementary information from both millimeter-wave radar data and visual camera images.

**Figure 1:**
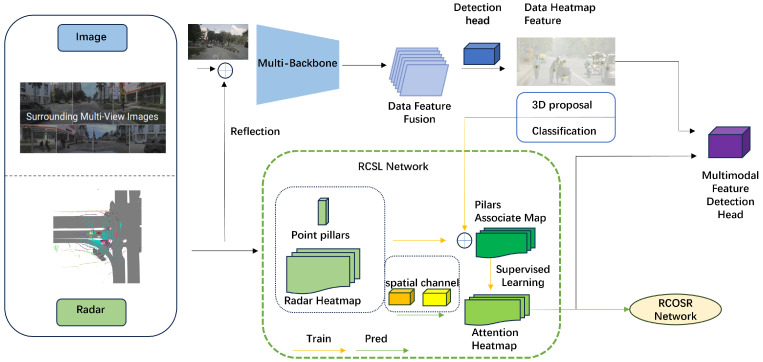
The overall architecture of the proposed RCRF network.

The overall architecture of the RCRFNet, depicted in Figure 1, consists of two parallel sub-networks for processing visual images and radar cloud data, respectively. Additionally, it features the following modules: the feature extraction backbone, the feature association and self-supervised learning module, the radar–camera open-set recognition module, and the multimodal feature detection head.

The feature extraction backbone: It extracts multilevel features from both radar point cloud data and visual images by projecting radar data onto pixel coordinates to obtain multidimensional data. Subsequently, a multi-layer fusion structure, consisting of upsampling and downsampling layers, is utilised to extract high-dimensional features and align data at various resolutions.

The feature association and self-supervised learning module: It innovatively selects high-fidelity radar–camera data pairs to generate self-supervised signals to boost the network’s performance by means of a frustum association process applied to the heatmaps of both radar data and visual images. Furthermore, spatial attention and channel attention mechanisms are introduced to enhance the heatmap association.

The radar–camera open-set recognition module: It is designed to improve object classification accuracy, especially for unknown categories.

The multimodal feature detection head: Finally, the fused multilevel features are linearly transformed to the same size for splicing, generating the final detection results through the constructed multimodal feature detector.

### 2.2. Feature Extraction Backbone and Heatmap Generation

**Figure 2:**
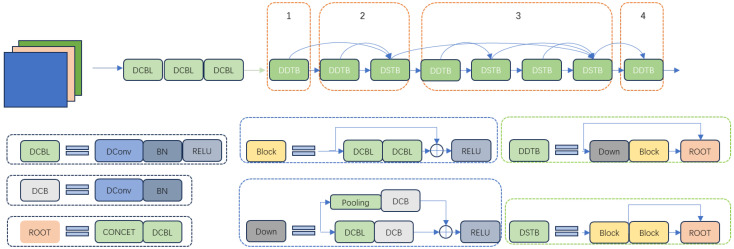
The designed DDLA feature extraction backbone. DConv denotes the deformable convolution layer.

We introduce a multi-layer fusion structure to merge different layers of radar and visual features, aiming to enhance the detector’s capability to detect targets across various scales. It is based on the deep layer aggregation (DLA) backbone from [32] and incorporates deformable convolution layers to capture the geometric deformations of targets. The designed Deformable DLA (DDLA) module is depicted in Figure 2.

The DLA34 network is a deep residual network [33] that utilizes a multi-layer hierarchical fusion structure. Its two-dimensional structure is made up of the hierarchical deep aggregation module, which preserves and combines feature channels by joining blocks from different stages into a tree. As a result, the network can integrate both deep and shallow feature information, producing more feature hierarchies and richer combinations. The adopted DLA34 in Figure 2 first goes through a base layer, and then the four tree hierarchies from level 1 to level 4. However, the existing DLA34 relies on standard n×n convolution kernels for feature extraction, which may not effectively capture geometric deformations of objects [34]. To address this deficiency, we incorporate deformable convolution layers into the DLA34 backbone, introducing additional parameter directions and expanding the learning range.

**Figure 3:**
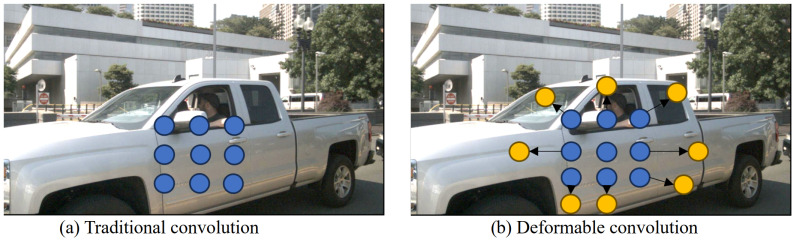
Contrast between (**a**) traditional convolution and (**b**) deformable convolution. The arrows in (**b**) indicate the additional parameter offsets introduced by deformable convolution.

Deformable convolution layers integrate extra offsets, enhancing the extraction of features for irregular objects. Figure 3 demonstrates the contrast between deformable convolution and traditional convolution, with arrows indicating the additional offsets introduced. This shows that deformable convolution can represent object features more efficiently. The deformable convolution can be expressed as follows:

(1) $$\beta \left(p_{0}\right)=\sum_{p_{n}\in R}w\left(p_{n}\right)\cdot \alpha \left(p_{0}+p_{n}+\Delta p_{n}\right)$$

where $w(p_{n})$ is the convolution layer weight, α is the feature map, $p_{0}$ is any point in the feature map, $p_{n}$ is the position vector of each point in the convolution kernel with respect to the centre, and $\Delta p_{n}$ denotes the positional offset between the feature map and the convolution kernel. A new offset has been introduced at each point, which is generated from the input feature map and another convolution kernel.

The image features extracted by the DDLA module are then fed into a tentative detection head to generate visual heatmaps for subsequent processing. Suppose the tentative detector obtains a series of target candidates belonging to *C* categories; then, the target candidates of the same category are utilised to generate a heatmap. Thus, we have

(2) $$H_{i}\left(m\left(P_{i}\right)\right)\in R^{\frac{H}{16}\times \frac{W}{16}\times 3},\;i=1,2,\cdots ,C$$

where $P_{i}$ denotes the set of target candidate centres corresponding to category *i*, and $m(P_{i})$ is a Gaussian kernel function applied to the set $P_{i}$. Let $P_{i}=\left\{p_{i,1},p_{i,2},\cdots ,p_{i,i_{N}}\right\}$, where $i_{N}$ is the number of target candidates belonging to category *i*. Then, $m(P_{i})$ can be expressed as follows:

(3) $$m\left(P_{i}\right)≜m\left(x\right)=\sum_{j=1}^{i_{N}}exp\left(-\frac{D^{2}\left(x,p_{i,j}\right)}{2\sigma_{i,j}^{2}}\right)$$

where x is the coordinate of any point on the *i*-th heatmap, $D(x,p_{i,j})$ is the Euclidean distance between the two points x and $p_{i,j}$, and $\sigma_{i,j}$ is the adaptive variance set by the target radius of $p_{i,j}$.

Next, we will introduce radar point cloud data filtering and heatmap generation. To address the issues of sparsity and low-resolution in radar point cloud data, we apply data filtering to enhance data density and suppress noise. Considering the targets’ manoeuvring movement, we utilise the extended Kalman filter (EKF) algorithm to preprocess radar point cloud data.

Let $\left(P_{x},P_{y}\right)$ and $\left(V_{x},V_{y}\right)$ denote the position and velocity of a target measured by the mmWave radar. The measurement model can be characterised by

(4) $$h\left(x\right)=\left[\begin{array}{c} R\\ \theta \\ V \end{array}\right]=\left[\begin{array}{c} \sqrt{P_{x}^{2}+P_{y}^{2}}\\ \arctan(P_{y}/P_{x})\\ \frac{P_{x}V_{x}+P_{y}V_{y}}{\sqrt{P_{x}^{2}+P_{y}^{2}}} \end{array}\right]$$

The Jacobian matrix for the measurement model is [35]

(5) $$J=\left[\begin{array}{cccc} \frac{P_{x}}{\sqrt{P_{x}^{2}+P_{y}^{2}}} & \frac{P_{y}}{\sqrt{P_{x}^{2}+P_{y}^{2}}} & 0 & 0\\ -\frac{P_{y}}{P_{x}^{2}+P_{y}^{2}} & \frac{P_{x}}{P_{x}^{2}+P_{y}^{2}} & 0 & 0\\ \frac{P_{y}\left(V_{x}P_{y}-V_{y}P_{x}\right)}{{\left(P_{x}^{2}+P_{y}^{2}\right)}^{3/2}} & \frac{P_{x}\left(V_{y}P_{x}-V_{x}P_{y}\right)}{{\left(P_{x}^{2}+P_{y}^{2}\right)}^{3/2}} & \frac{P_{x}}{\sqrt{P_{x}^{2}+P_{y}^{2}}} & \frac{P_{y}}{\sqrt{P_{x}^{2}+P_{y}^{2}}} \end{array}\right]$$

In practice, we take the weighted sum of the current frame data and the Kalman filter data as the preprocessing results. Furthermore, the radar heatmap is generated using Gaussian kernels with the preprocessed point cloud as the centre and a fixed value as the radius. Each point cloud has three channels corresponding to range, azimuth angle, and radial velocity, respectively. Additionally, the point cloud is extended into a pillar with a preset value to address the association deficiency caused by missing height information.

### 2.3. Radar–Camera Association and Self-Supervised Learning

The visual camera and mmWave radar sensors can both offer complimentary data about the target. Visual images provide high resolution and can capture detailed features such as geometry, contours, and texture, but they are sensitive to variations in lighting conditions. Conversely, despite lower data resolution and angular accuracy, radar point cloud data can provide physical attribute information about the target, such as speed, angle, and distance, regardless of the lighting.

To leverage the complementary advantages of both sensors, this paper adopts a process where high-resolution visual image features serve as a reference, and point cloud data with high spatial location matching are then selected for correlation and fusion. In essence, correlated data obtained under favourable lighting conditions are chosen to generate self-supervised signals. Our method aims to enhance the network’s feature learning and representation capabilities by leveraging the inherent strengths of both sensor modalities.

**Figure 4:**
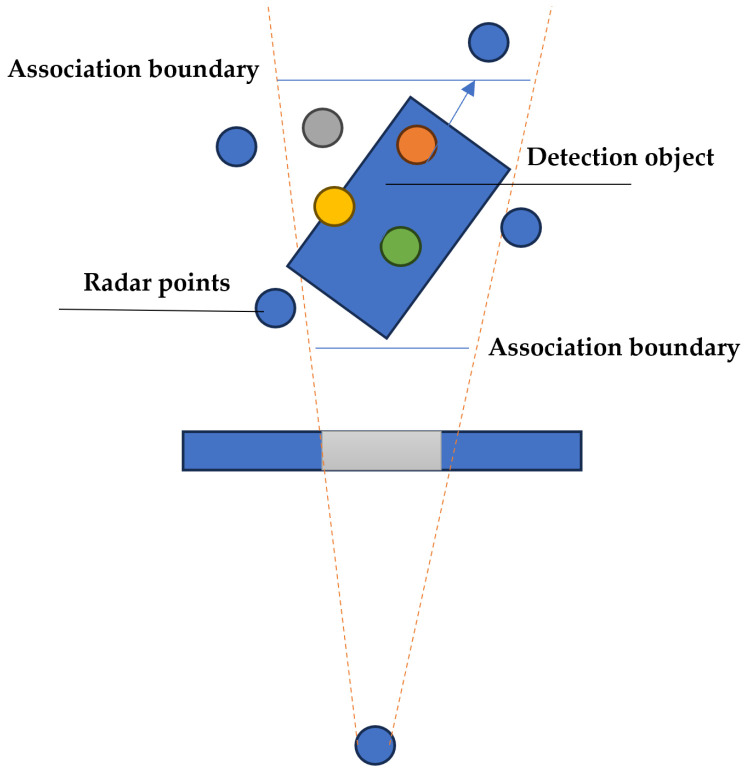
The frustum association approach in the bird’s eye view.

Specifically, the frustum association approach is utilised to correlate the radar point cloud with visual images, generating the self-supervised signal for training, as depicted in Figure 4. The target’s 3D bounding box is transformed into a frustum coordinate system, with its centre and orientation angle denoted as $\left(O_{X},O_{Y},O_{Z},\phi \right)$. The orientation of the target in the bird’s eye view is also indicated by an arrow in Figure 4. The association between the radar point cloud $\left(P_{X},P_{Y},P_{Z}\right)$ and the target’s bounding box is achieved by calculating the following Euclidean distance:

(6) $$\rho_{i}=\sqrt{{\left(P_{X}^{2}-O_{X}\right)}^{2}+{\left(P_{Y}^{2}-O_{Y}\right)}^{2}+{\left(P_{Z}^{2}-O_{Z}\right)}^{2}}$$

**Figure 5:**
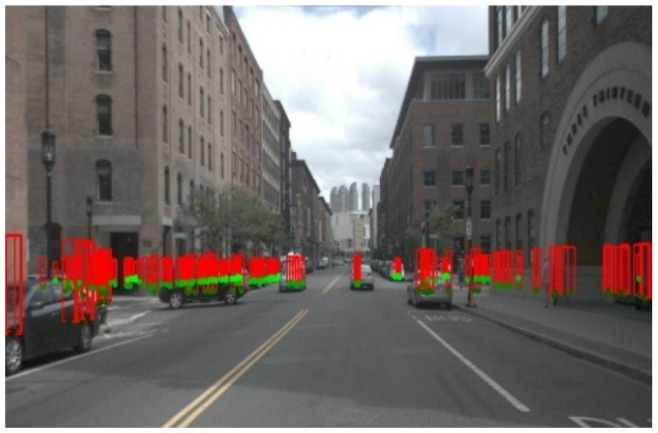
An example of the frustum association result.

The point cloud with the lowest Euclidean distance is selected, and its heatmap will be concatenated with the image heatmap to generate the final detection results. An example of the frustum association result is shown in Figure 5. We can see that the radar point cloud is well matched with the target in the image. Here, the radar point cloud is shown in the pillar format.

It is important to emphasise that the frustum association approach heavily depends on visual detection results. When visual information is unreliable, it struggles to accurately correlate millimeter-wave radar point clouds.

Next, the mean squared error (MSE) loss between the radar heatmap and the frustum-associated heatmap is utilised for self-supervised learning to accelerate the convergence speed of the model:

(7) $$L_{H}=\frac{\sum_{x=1}^{H}\sum_{y=1}^{W}{\left(H_{o}\left(x,y\right)-H_{a}\left(x,y\right)\right)}^{2}}{H\times W}$$

where $H_{o}$ and $H_{a}$ are the radar heatmaps generated by all point clouds and the associated point cloud, respectively. In the prediction phase, the associated radar heatmap is directly concatenated with the image heatmap and fed into the detection head to generate the final results.

**Figure 6:**
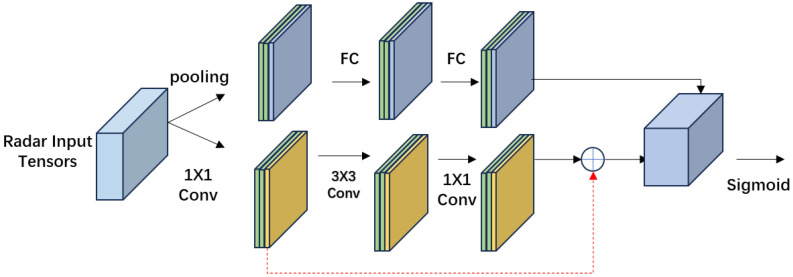
The attention module for enhancing the quality of the association heatmap.

Additionally, to enhance the quality of the associated radar heatmap, spatial attention and channel attention mechanisms are introduced, as shown in Figure 6. First, features are compressed in the spatial dimension using global average pooling to gain a larger receptive field. Subsequently, normalised weights are obtained through a fully connected layer, activation function, and normalisation operation. Finally, these weights are used to emphasise the importance of each feature channel, resulting in the attention-enhanced associated point cloud heatmap. Moreover, the radar point cloud features are used to filter noise and outliers, thereby reducing false positives during detection.

### 2.4. Radar–Camera Open-Set Recognition Module

For the visual heatmap generation in Equation (2), it is assumed that the targets to be detected belong to *C* predefined categories. However, targets in real-world scenarios are diverse, and environments are complex. Thus, it is challenging to enumerate all possible target categories in advance. When a target to be detected does not fall within one of the *C* predefined categories, target detection performance may significantly decrease. To address this issue and improve the approach’s performance in real-world applications, recently developed open-set techniques are introduced, and a radar–camera open-set recognition (RCOSR) module is designed.

**Figure 7:**
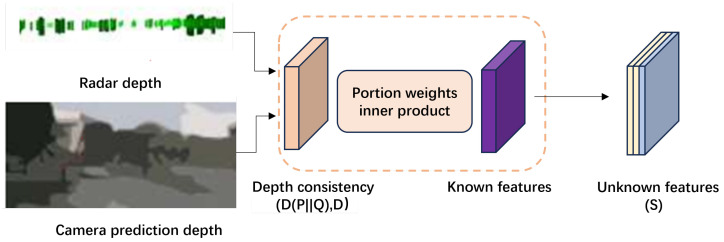
Radar–camera open-set recognition module.

The RCOSR leverages the complementary strengths of both sensors to improve the recognition accuracy of unknown class targets. Visual images, with their high resolution and detailed target information, enable highly confident target category predictions based on visual features. However, they are vulnerable to weather and lighting conditions, and the depth information they provide is often ambiguous. In contrast, radar provides more accurate depth information than visual images [36]. Therefore, we propose a novel depth-information-constrained feature aggregation method for open-set recognition, as depicted in Figure 7. Specifically, we calculate the global and local depth consistencies between the radar point cloud and visual images, which are then multiplied with visual image features to construct known target confidence levels, thus achieving unknown target detection.

The global depth consistency is quantified using the Kullback–Leibler (KL) divergence between the radar depth image $d_{R}\left(x,y\right)$ and the camera depth image $d_{C}\left(x,y\right)$, as shown below:

(8) $$D(P||Q)≜D(d_{R}\left|\right|d_{C})=\sum d_{R}\left(x,y\right)log_{2}\frac{d_{R}\left(x,y\right)}{d_{C}\left(x,y\right)}.$$

The local depth consistency D(x,y) at the coordinate (x,y) is calculated using the following formula:

(9) $$D\left(x,y\right)=1-softmax\left(abs(d_{R}\left(x,y\right)-d_{C}\left(x,y\right))\right).$$

Additionally, considering that the occurrence probabilities of each target category in real-world scenarios may vary significantly and affect the discrimination of unknown classes, a new weight $\sigma_{i}$ measuring the occurrence probability is introduced. Finally, the weighted confidence level of known class targets is determined:

(10) $$S\left(x,y\right)=\frac{1}{\left|C\right|}\cdot D\left(P||Q\right)\cdot \sum_{i=1}^{C}\sigma_{i}\cdot D\left(x,y\right)).$$

A target candidate with $S\left(x,y\right)\leq S_{t}$ is classified as an unknown class target, where $S_{t}$ is a threshold set according to the confidence probability.

### 2.5. Multimodal Feature Detection Head

**Figure 8:**
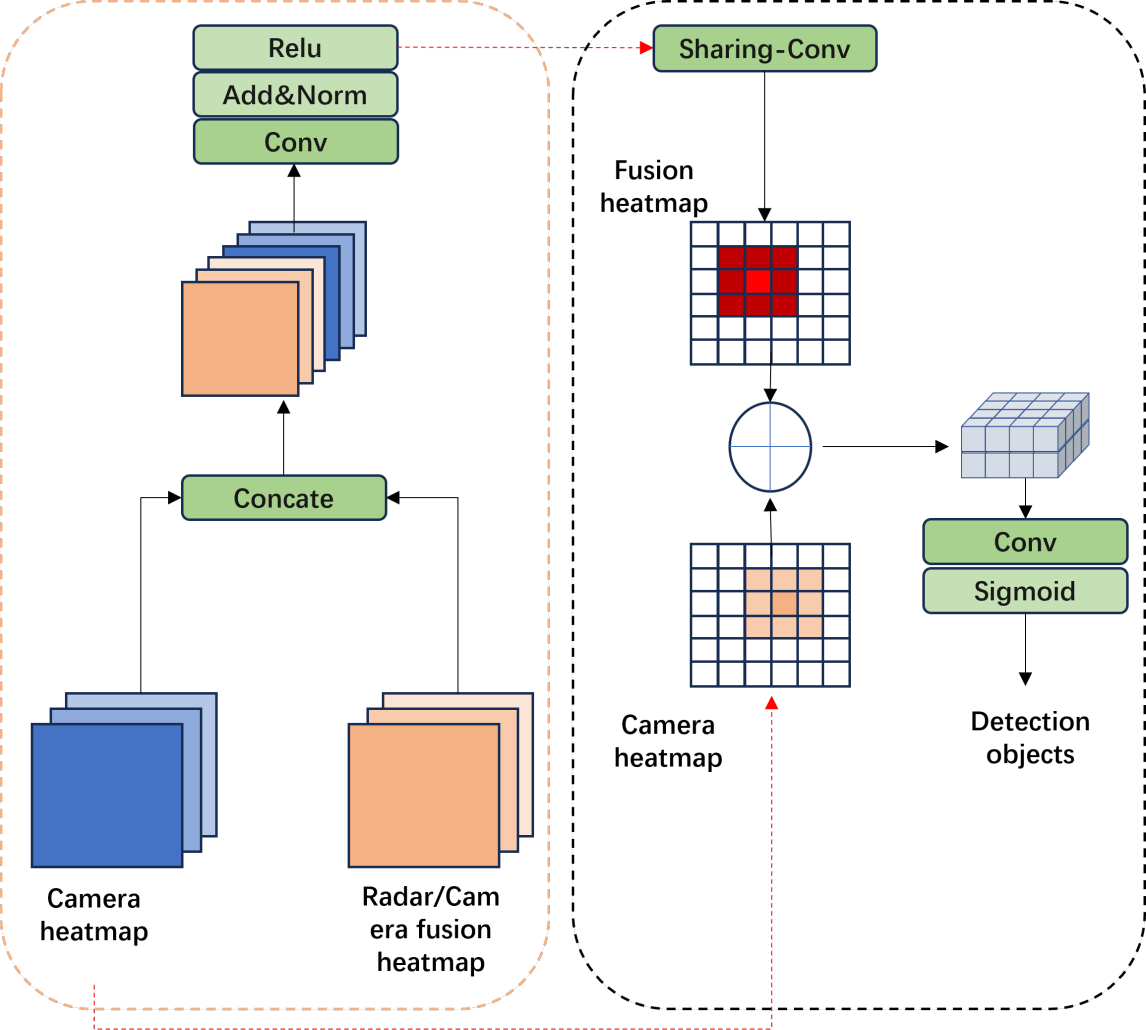
Multimodal feature detection head.

The multimodal feature detection head comprises two stages. The first stage, comprising a convolution layer and an activation function, is designed to generate target proposals for radar–camera association. The second stage generates the final fusion features through point-to-point splicing matching between the fusion heatmap and the camera heatmap. The structure of the detection head is illustrated in Figure 8. This two-stage design ensures more reliable detection.

The loss function of the proposed model has two parts: the focal loss and the binary cross-entropy (BCE) loss, which can be expressed as

(11) $$\begin{array}{cc} L_{total} & =L_{pre}+L_{fusion}\\ \; & =\left(L_{pre,cls}+L_{pre,reg}\right)+\left(L_{fusion,cls}+L_{fusion,reg}\right) \end{array}$$

The focal loss $L_{pre,cls}$ and $L_{fusion,cls}$ are associated with category information, while the BCE loss $L_{pre,reg}$ and $L_{fusion,reg}$ pertain to size information. This combination allows the model to effectively learn and optimise for both category and size information.

## 3. Experiments and Results

The experiments are divided into three sections: Section 3.1 presents the overall detection performance of the proposed approach, Section 3.2 focuses on robustness analysis, and Section 3.3 primarily verifies the open-set performance.

For comparison, three state-of-the-art (SOTA) detection algorithms for visual images—Mono3D [37], CenterNet [38], and FCOS3D [39]—as well as the latest fusion detection method, CenterFusion [18], are selected as benchmarks. For the open-set recognition module, the traditional Openmax method is chosen for comparison because current mainstream radar–camera fusion detection methods rarely address the open-set issue.

We used the nuScenes dataset [40] for the experiments. This dataset comprises data from mmWave radar, cameras, and other sensors, as detailed in Table 1. We selected data from over 1000 scenes in various environments and lighting conditions. Each scene contains 40 keyframes and 20 s of data.

The experiments were conducted on an Ubuntu 18.4 system, equipped with four GeForce RTX 4080 GPUs (NVIDIA, Santa Clara, CA, USA), each with 24 GB of RAM. The Adam optimizer was used to iteratively update the network weights, with a weight decay rate set to 0.0001. Following a step learning strategy, the learning rate was halved every 20 epochs. A total of 50 epochs of training were performed. The training parameter settings are summarised in Table 2. Preprocessing provided by the nuScenes official website was applied to the dataset during the experiments.

To evaluate detection accuracy, we use the average precision (AP) metric, where a match is determined by thresholding the two-dimensional centre distance on the ground plane. The mean average precision (mAP) is calculated as follows:

(12) $$\begin{array}{c} \mathrm{mAP}=\frac{1}{\left|C||M\right|}\sum_{c\in C}\sum_{m\in M}{\mathrm{AP}}_{c,m} \end{array}$$

where *C* denotes the number of predicted classes and *M* represents the distance threshold between the predicted bounding box and the ground truth. In this study, we set the threshold values to M=0.5,1,2,4 m.

Additionally, the experiments calculate five true-positive (TP) metrics: average translation error (ATE), average scale error (ASE), average orientation error (AOE), average velocity error (AVE), and average attribute error (AAE). For each of these metrics, the mean true positive (mTP) for all classes can be derived as follows:

(13) $$\begin{array}{c} \mathrm{mTP}\left(x\right)=\frac{1}{\left|C\right|}\sum_{c\in C}\left(1-min\left\{1,x\right\}\right),x\in \left\{\mathrm{ATE},\mathrm{ASE},\mathrm{AOE},\mathrm{AVE},\mathrm{AAE}\right\} \end{array}$$

Following this, the nuScenes detection score (NDS) is computed as

(14) $$\begin{array}{c} \mathrm{NDS}=\frac{1}{10}\left[5\times \mathrm{mAP}+\sum_{x}\left(1-min\{1,\mathrm{mTP}(x)\}\right)\right] \end{array}$$

This metric effectively encapsulates various aspects of the detection task, including velocity and attribute estimation.

### 3.1. Overall Detection Performance

The comparison results of our approach with SOTA algorithms are presented in Table 3, where all the aforementioned metrics are evaluated. Among these metrics, NDS and mAP are the two most representative, with higher values indicating better performance. As shown in Table 3, our approach achieves the highest NDS and mAP scores. Specifically, the NDS improves to 0.46, representing a 2.5% relative improvement over CenterFusion. Additionally, the AOE and AVE show significant relative improvements of 12.6% and 9.8%, respectively. Our approach incorporates radar information through the RCSL module, leading to a substantial enhancement in the accuracy of target speed estimation, as reflected in the AVE metrics. In summary, the proposed method effectively fuses mmWave radar point cloud data with visual images, resulting in improved overall detection performance.

**Figure 9:**
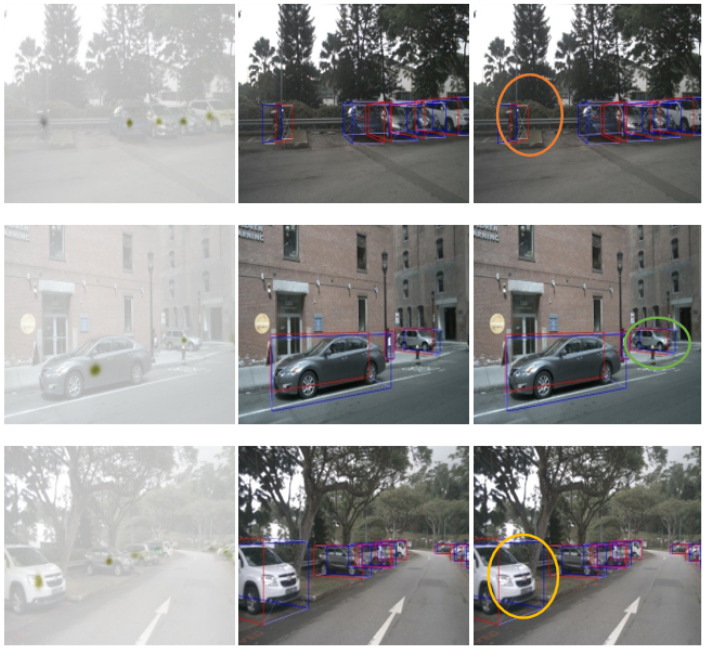
Results of our approach under harsh environmental conditions.

Figure 9 illustrates several visualisation results of our proposed approach. We selected three representative frames from the test data, which include scenarios with pedestrian interference, target occlusion, and multi-scale small objects. The target centre, ground truth, and detection results for these frames are displayed from left to right. Our method demonstrates notable proficiency in detecting and identifying distant objects (marked in green), occluded objects (marked in yellow), and obstacles (marked in orange).

**Figure 10:**
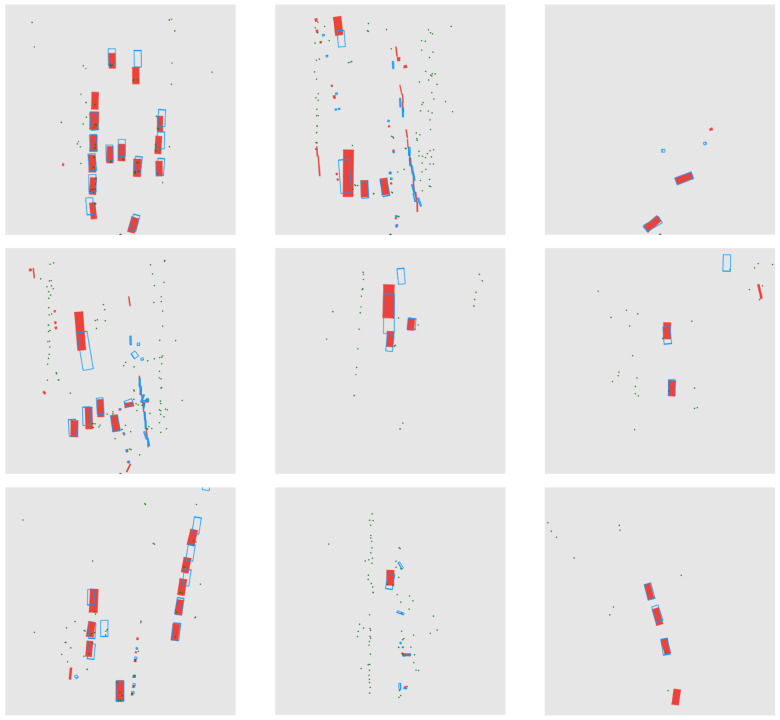
BEV plots of detection results.

For autonomous driving, target detection results are occasionally converted into bird’s eye view (BEV) to aid in downstream planning and control tasks. Figure 10 shows several BEV plots of detection results in typical scenarios. These plots demonstrate that RCRFNet delivers exceptional detection results by fusing radar and camera data.

### 3.2. Robustness Performance Analysis

To validate the robustness of the proposed method, we conducted a comparative analysis under various visibility conditions. In this section, experiments were performed with images of four different visibility levels, while keeping other parameter settings unchanged. This setup simulates different weather conditions. Table 4 presents the experimental results for CenterFusion and our RCRFNet. In this table, the maximum loss value, calculated as the difference between the performance index of the highest visibility image and that of the lowest visibility image, is used as the robustness criterion. It can be observed that the RCRFNet exhibits a lower maximum loss value for NDS, mAOE, mAVE, and mAP, indicating higher robustness under varying weather conditions.

**Figure 11:**
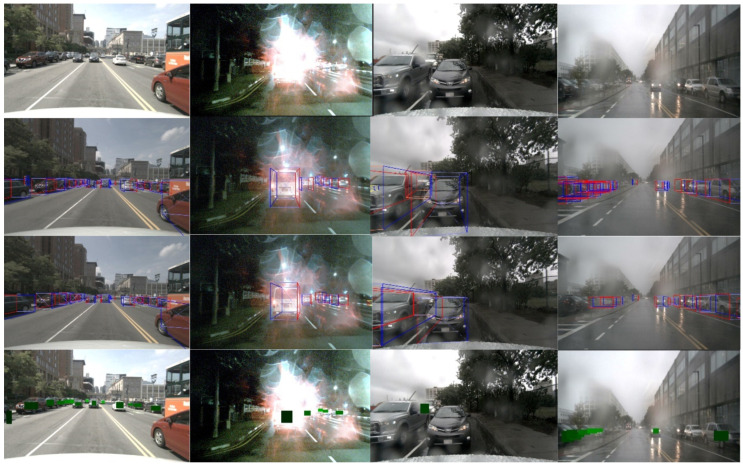
Results of our approach under harsh environmental conditions.

Table 5 details the performance of CenterFusion and the RCRFNet method across four visibility levels: 0–40%, 40–60%, 60–80%, and 80–100%. In these fine-grained comparative experiments, the training data volume is significantly reduced due to data screening, posing additional challenges for detection models. Despite this, our method outperforms CenterFusion, thanks to the implementation of various new processing modules that effectively integrate the complementary strengths of radar point cloud data and camera image data. Figure 11 displays several detection results of the RCRFNet method under harsh environmental conditions. From top to bottom, the images are presented as the original image, ground truth, detection results, and image with the object’s depth information.

### 3.3. Open-Set Performance Analysis

In this subsection, we aim to verify the effectiveness of the RCOSR module proposed in this paper. We divide the dataset categories into known and unknown categories. During training, only the known categories are used, and the trained model is then applied in open-set recognition experiments. A comparison with the Openmax method under two different visibility levels is shown in Table 6. To ensure a fair comparison, the number of training iterations is set to 30, while other parameters remain unchanged. Table 7 presents detailed performance results for the closed-set categories of Openmax and the RCRFNet method. The experimental results on the nuScenes dataset demonstrate that the radar–vision fusion open-set recognition method proposed in this paper achieves better detection accuracy. These findings confirm the effectiveness of the RCOSR module to a certain extent.

## 4. Ablation Study

This section describes the ablation experiments on the NuScenes dataset, using CenterFusion as our baseline to validate the effectiveness of each module: deformable convolution layers, RCSL, and RCOSR.

In the experiments, we incrementally included the modules of deformable convolution layers, RCSL, and RCOSR into our model. The results are summarised in Table 8. The findings indicate that the deformable convolution layers module improves NDS compared to baseline. The RCSL module, which primarily enhances robustness, improves the mAAE and mAOE metrics. The RCOSR module, by computing a contrastive loss between radar depth information and the depth predicted from images, corrects the depth prediction derived from images, significantly enhancing both mAP and NDS. Notably, the NDS index shows an improvement of 8.9%, demonstrating the effectiveness of incorporating radar depth information to enhance detection performance.

Furthermore, the comparisons of the model parameter size and the NDS metric between our approach and SOTA methods are listed in Table 9. It can be seen that the proposed RCRFNet has only a 4.1% model size increase compared to CenterFusion but has achieved a 2.5% improvement in the NDS metric.

**Figure 12:**
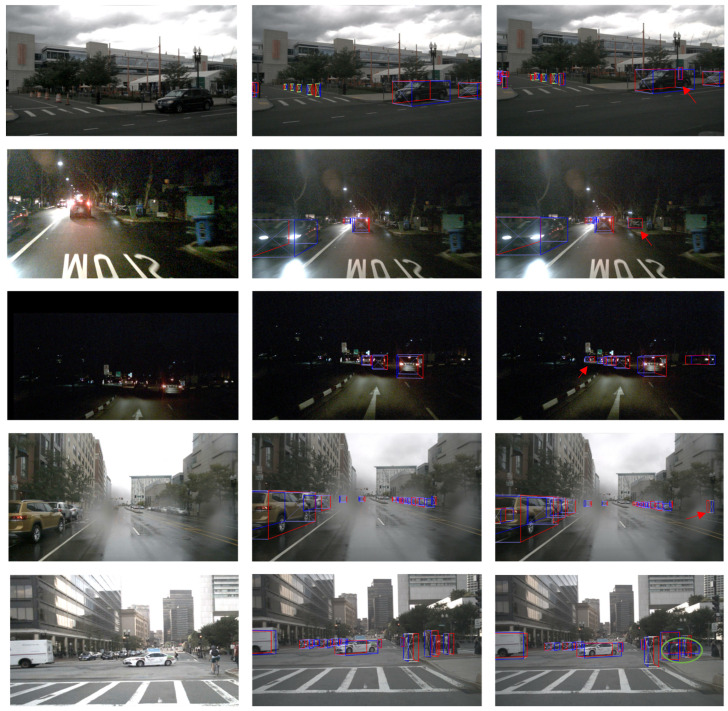
Qualitative comparison between our approach and CenterFusion under various scene conditions. For each row, the images from left to right are the raw image, the detection result of CenterFusion, and the result of our approach.

To demonstrate the superior performance of our approach compared to the CenterFusion method under various scene conditions, Figure 12 presents detection results for scenarios including occluded obstacles, small objects in low-light conditions, long-distance observations, and lens-contaminated scenes. It is evident that our approach is more effective in detecting distant objects and handling scenes with occlusions and lens contamination.

## 5. Conclusions

To address the limitations of current mainstream methods in robust target detection under low visual visibility and the challenge of effectively handling unknown class targets, this paper proposes a new fusion method that combines mmWave radar point cloud data with visual images. The method introduces an adaptive correlation module for enhanced robustness and incorporates uncertainty calculation for the recognition of unknown category targets. Experimental results on the nuScenes dataset show that the proposed RCRFNet method outperforms SOTA methods, particularly in low-visibility scenes. Although the RCRFNet method is currently designed for data within the field of view, future research will explore its application in bird’s eye view, potentially further improving target detection and classification performance.

## Figures and Tables

**Table 1:** Dataset details.

Tensors	Information
Camera	50 m, 12 Hz
Radar	250 m, 13 Hz
LiDAR	70 m, 20 Hz
GPS and IMU	RTK ap: 20 mm, 1000 Hz

**Table 2:** Training parameter settings.

Parameter	Epoch	Batch	Momenta	Learning Rate
Value	50	64	0.922	0.0001

**Table 3:** Comparison results with SOTA algorithms on the nuScenes dataset. ↑ indicates that higher is better and ↓ indicates that lower is better.

Method	Modalities	NDS↑	mAP↑	Error (↓)				
				mATE	mASE	mAOE	mAVE	mAAE
Mono3D	C	0.429	0.366	0.642	0.252	0.523	1.591	0.119
CenterNet	C	0.400	0.338	0.658	0.255	0.629	1.629	0.142
FCOS3D	C	0.428	0.358	0.690	0.249	0.452	1.434	0.124
CenterFusion	R + C	0.449	0.326	0.631	0.261	0.516	0.614	0.115
RCRFNet	R + C	0.460	0.335	0.658	0.270	0.451	0.554	0.145

CF: CenterFusion; RF: RCRFNet.

**Table 4:** Comparison of the maximum loss value under varying image visibility levels.

Metrics	CenterFusion	RCRFNet
NDS	0.0207	0.0168
mAOE	0.0379	0.0152
mAVE	0.0998	0.0820
mAP	0.0296	0.0094

**Table 5:** Performance for four different image visibility levels. ↑ indicates that higher is better and ↓ indicates that lower is better.

Visibility	0–40%	40–60%	60–80%	80–100%
NDS↑ (CF)	0.3639	0.3765	0.3795	0.3846
NDS↑ (RF)	0.4414	0.4419	0.4490	0.4582
mAOE↓ (CF)	0.5108	0.4824	0.4691	0.4729
mAOE↓ (RF)	0.4459	0.4560	0.4475	0.4307
mAVE↓ (CF)	1.3009	1.2472	1.2598	1.2011
mAVE↓ (RF)	0.6395	0.5933	0.5797	0.5575
mAP↑ (CF)	0.2707	0.2904	0.2940	0.3003
mAP↑ (RF)	0.3191	0.3130	0.3227	0.3285

CF: CenterFusion; RF: RCRFNet.

**Table 6:** Open-set accuracy comparison.

Method	mAP	
Visibility	40–70%	70–100%
Openmax	0.387	0.415
RCRFNet	0.405	0.427

**Table 7:** Comparison details for closed-set categories.

Method	Car	Truck	Bus	Trailer	Vehicle	Ped.	Moto	Bicycle	Traffic
Openmax	0.455	0.209	0.295	0.089	0.020	0.338	0.269	0.172	0.522
RCRFNet	0.466	0.223	0.314	0.105	0.021	0.346	0.259	0.175	0.520

**Table 8:** Ablation experiments for each module. ↑ indicates that higher is better and ↓ indicates that lower is better.

Method	Rad	DC	RL	RR	NDS↑	mAP↑	Error (↓)				
							mATE	mASE	mAOE	mAVE	mAAE
Ours	✔	✔	-	-	0.451	0.326	0.674	0.267	0.445	0.555	0.154
Ours	✔	✔	✔	-	0.456	0.328	0.672	0.266	0.435	0.570	0.142
Ours	✔	✔	✔	✔	0.460	0.335	0.658	0.270	0.451	0.554	0.145

Rad: radar; DC: deformable convolution; RL: RCSL; RR: RCOSR.

**Table 9:** Comparisons of model size and NDS metric.

Method	Parameters (M)	NDS (%)
FCOS3D	127.6	41.5
DETR3D	613.2	42.2
CenterNet (Hourglass)	730.4	40.0
CenterNet (DLA)	241.6	32.8
CenterFusion (DLA)	248.9	44.9
RCRFNet (DLA)	258.7	46.0

## Data Availability

The data presented in this study are openly available in [nuScenes] at [https://doi.org/10.1109/CVPR42600.2020.01164], reference number [40].
